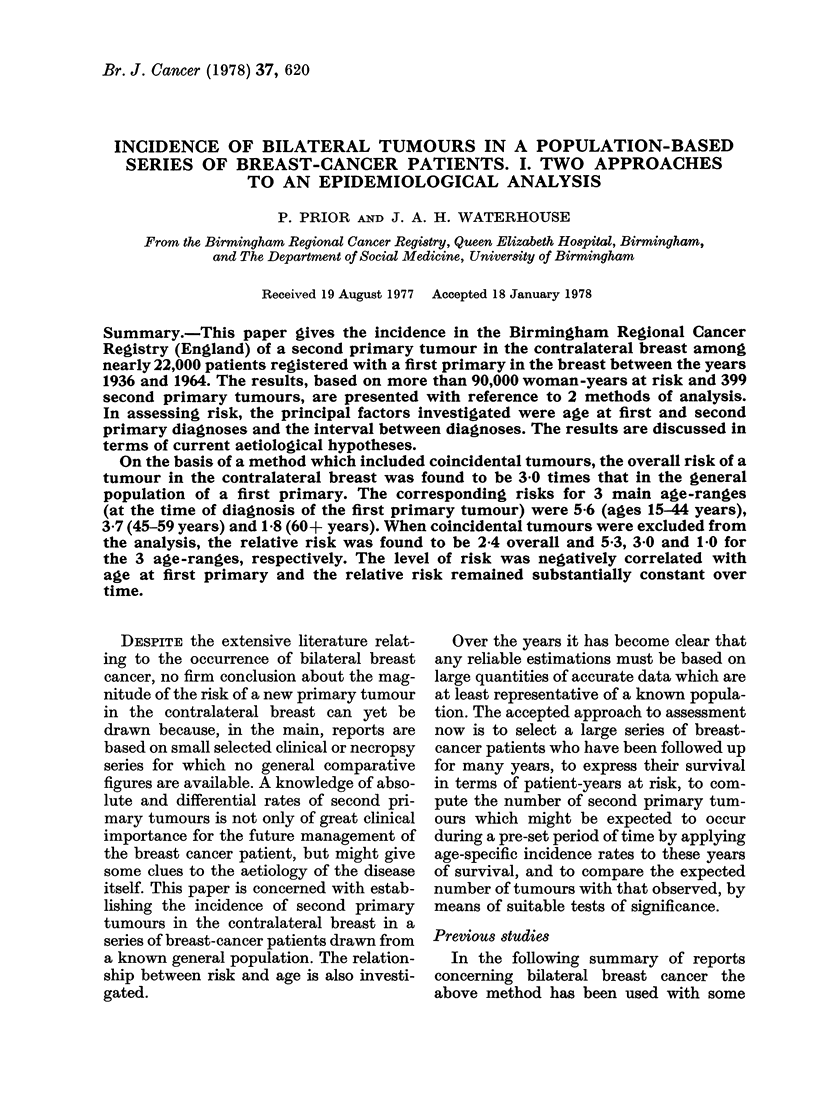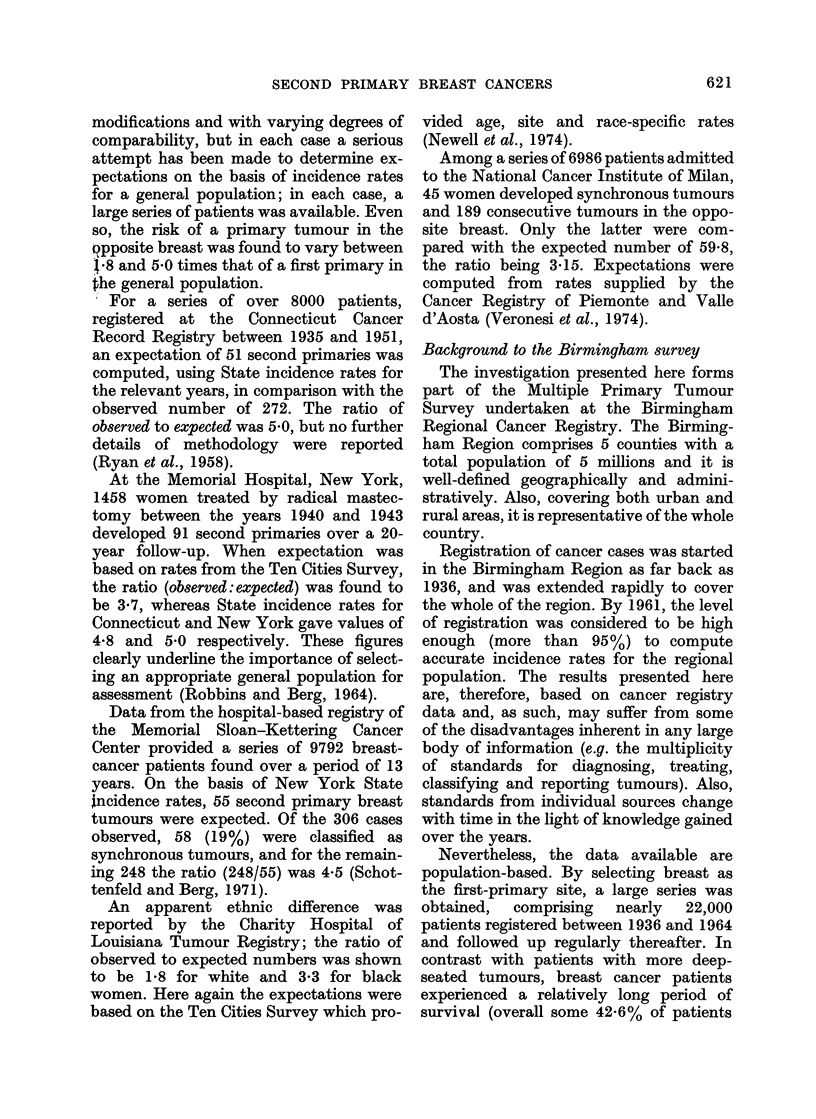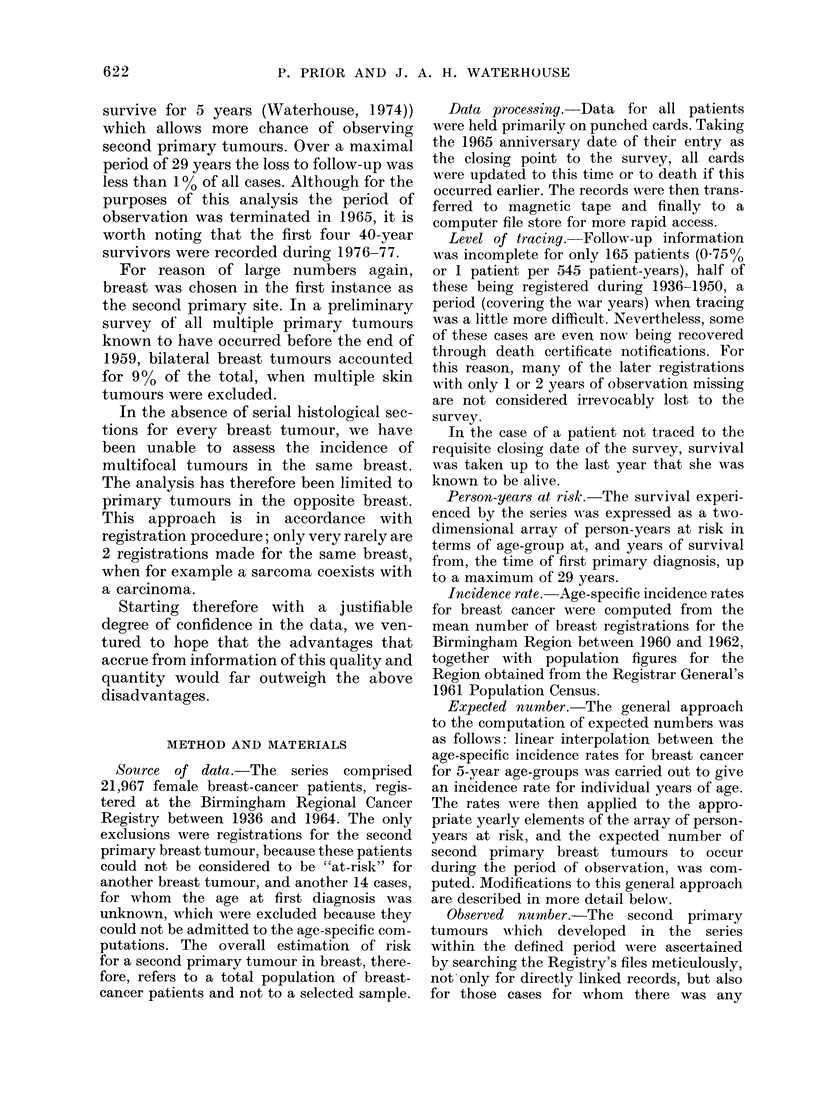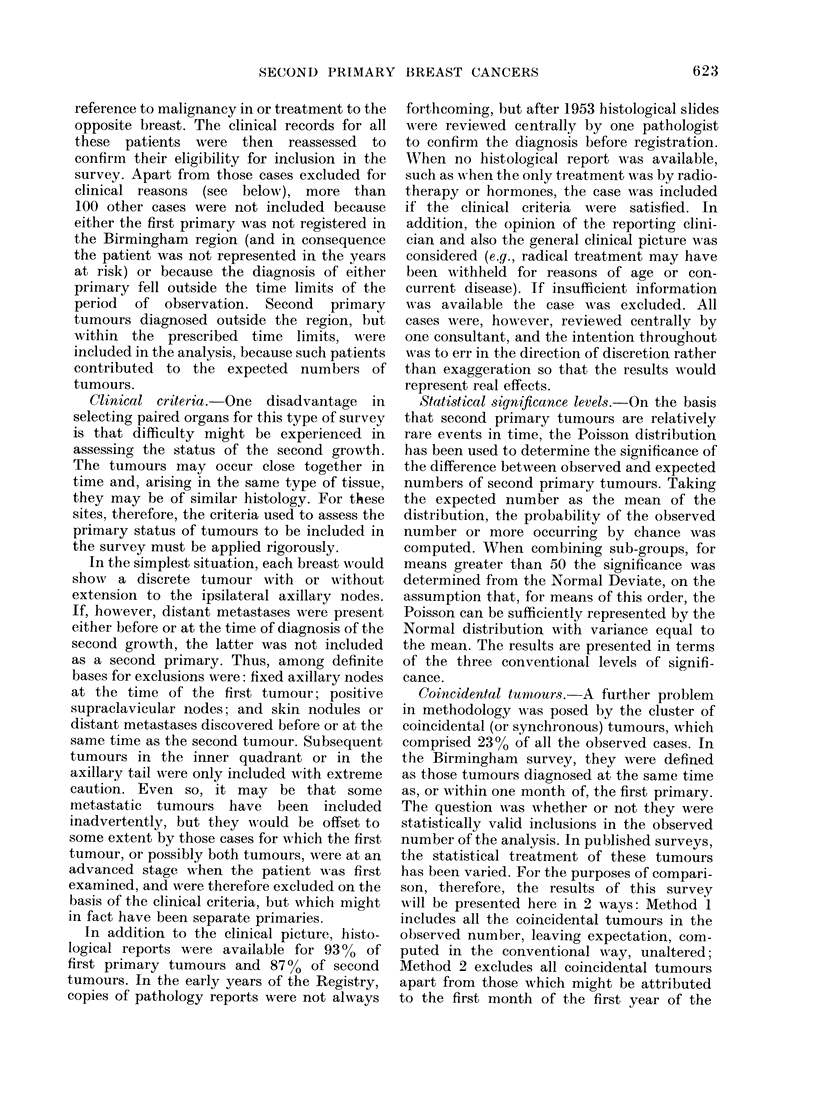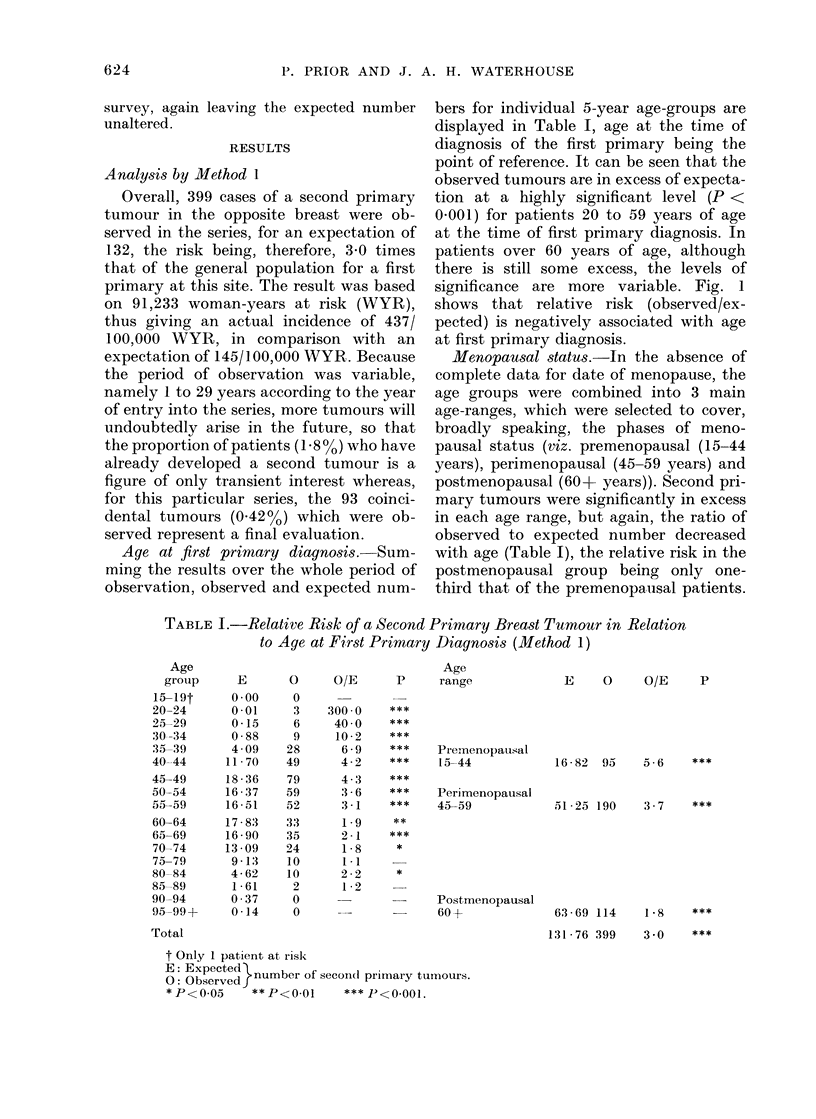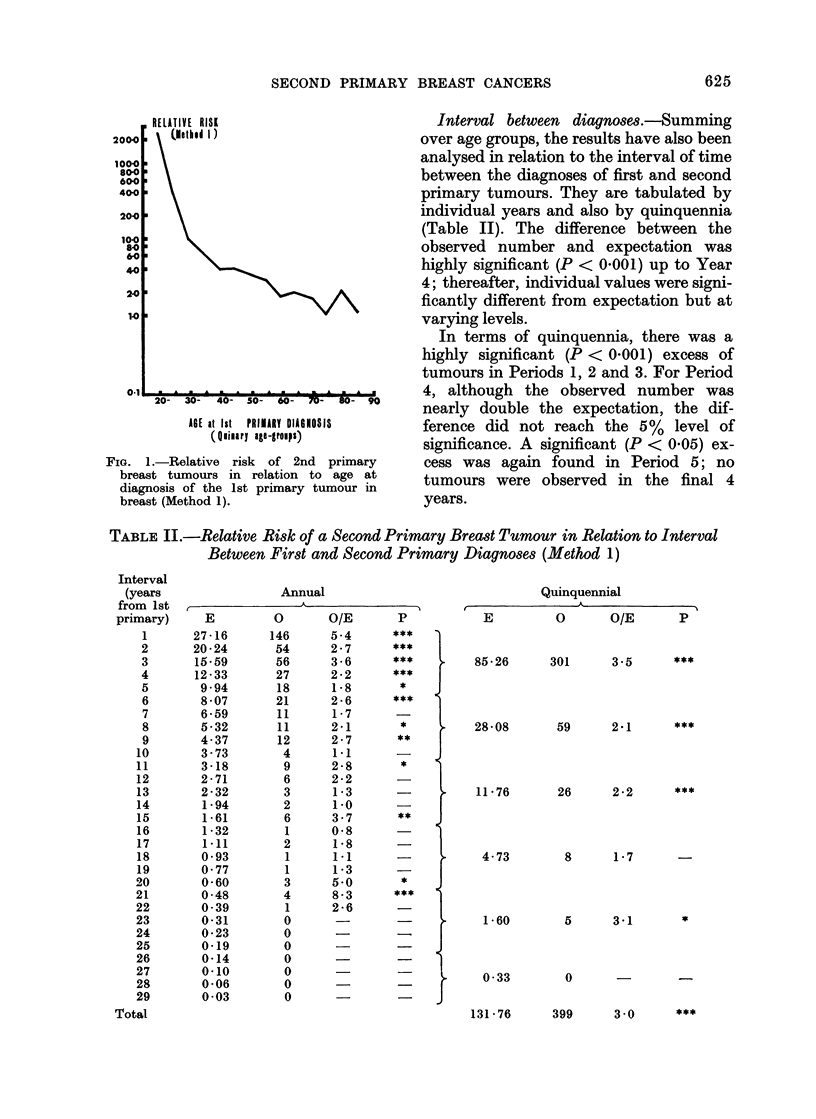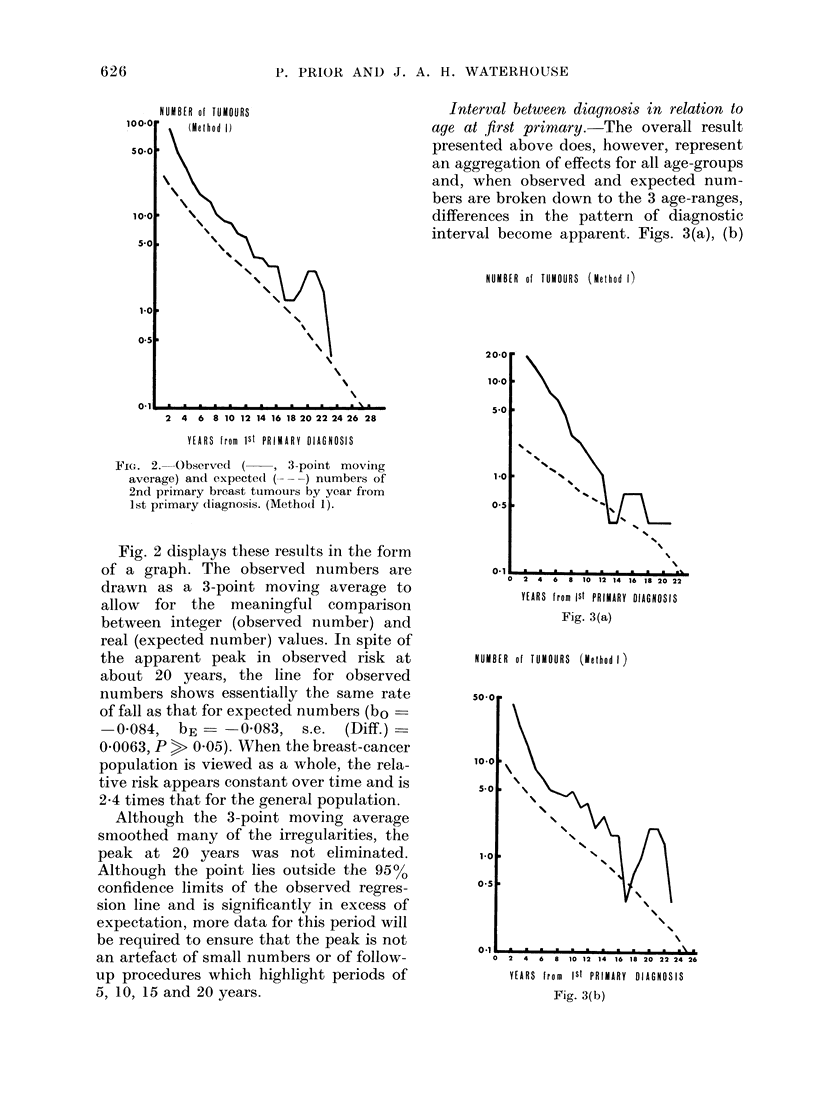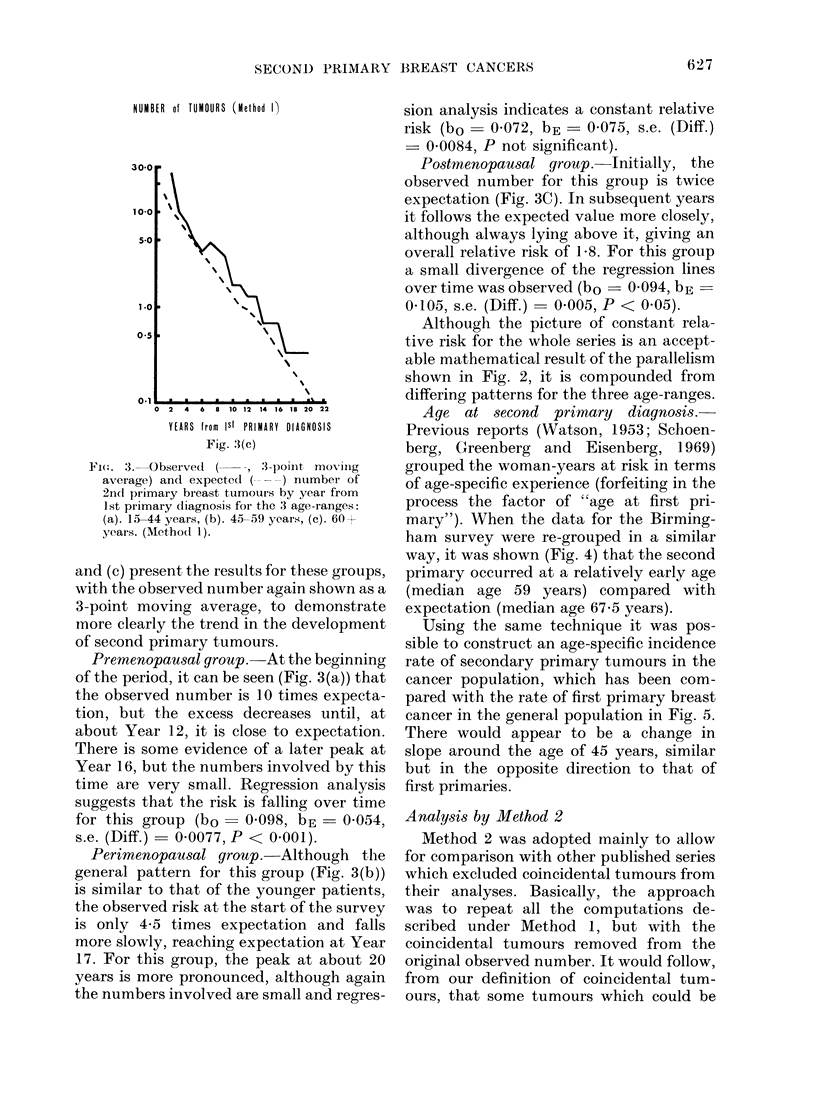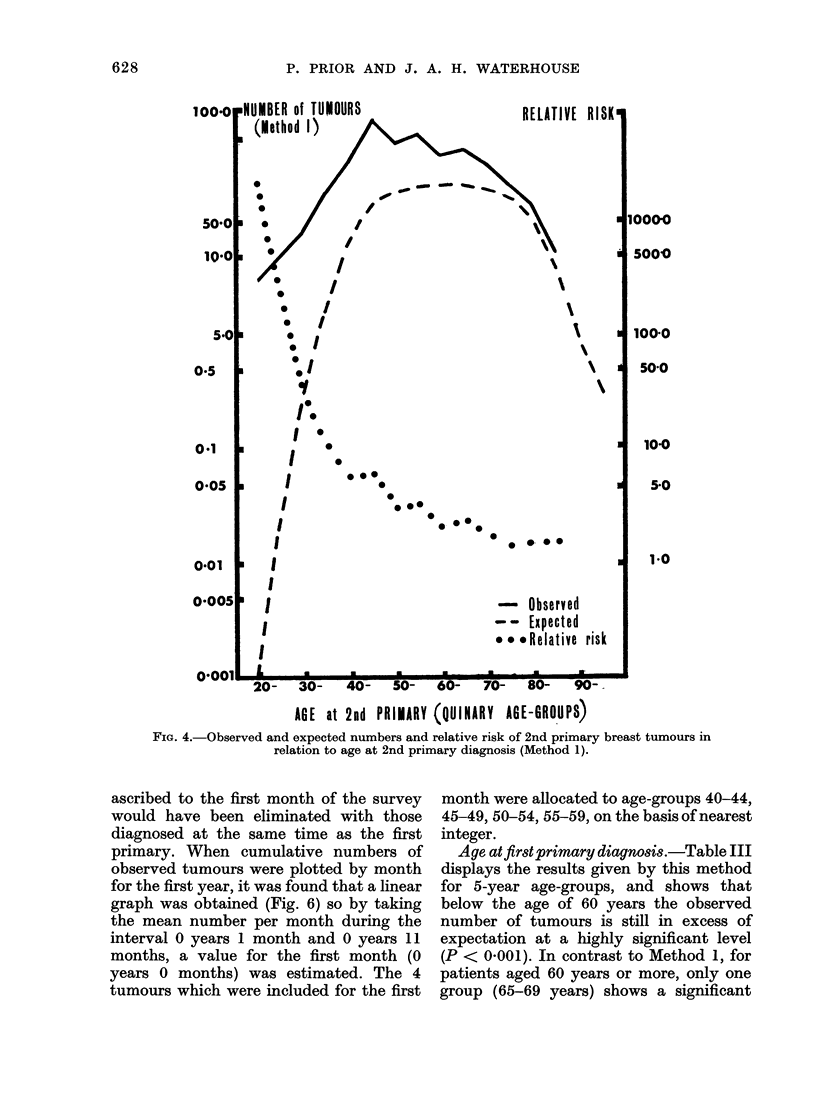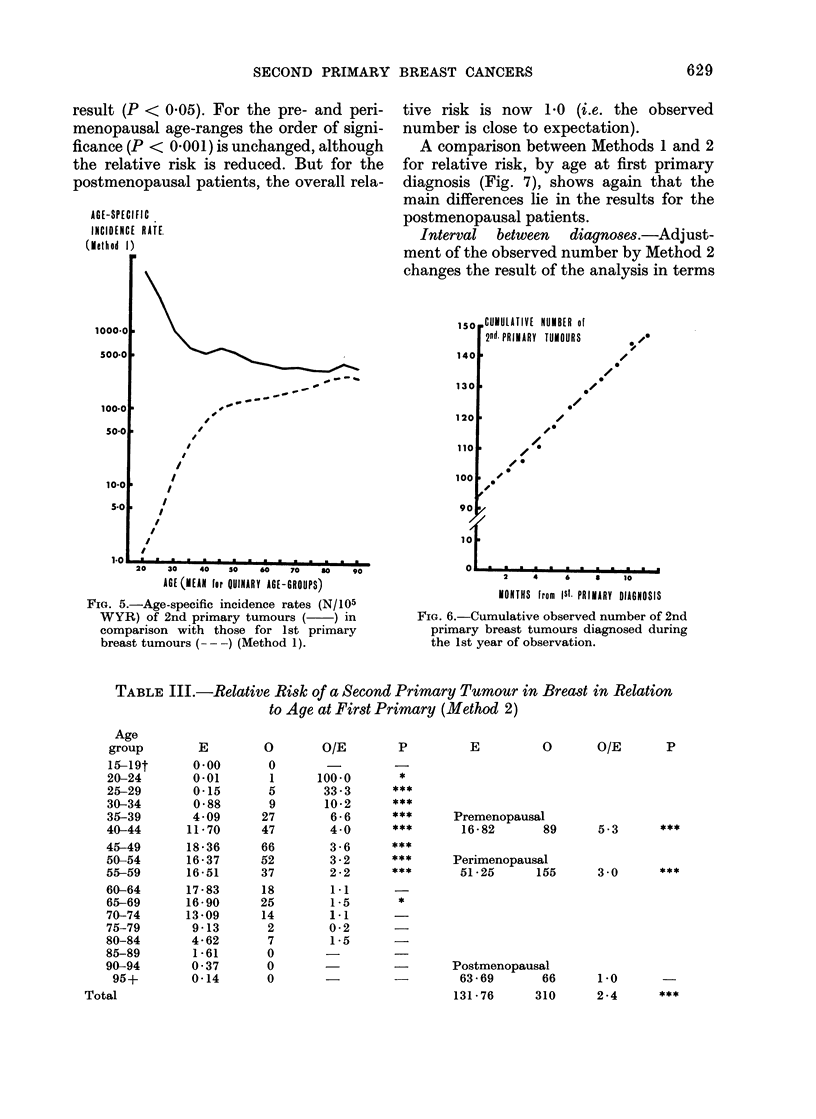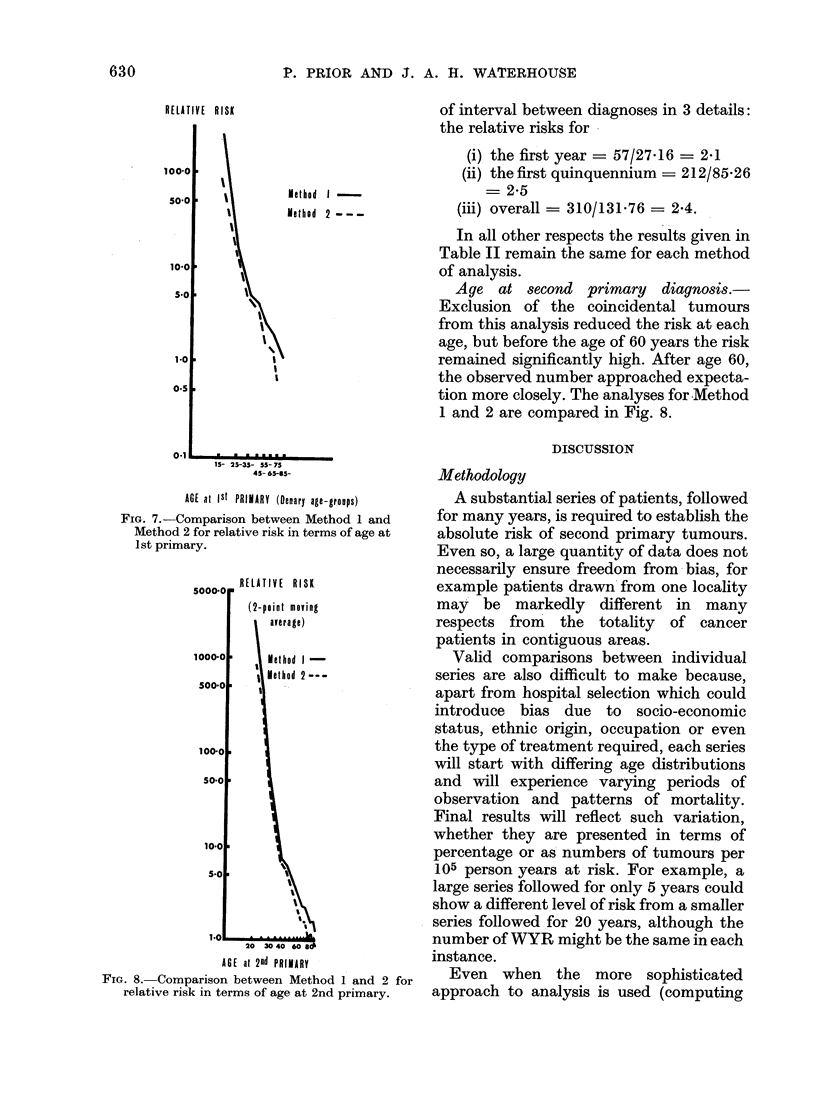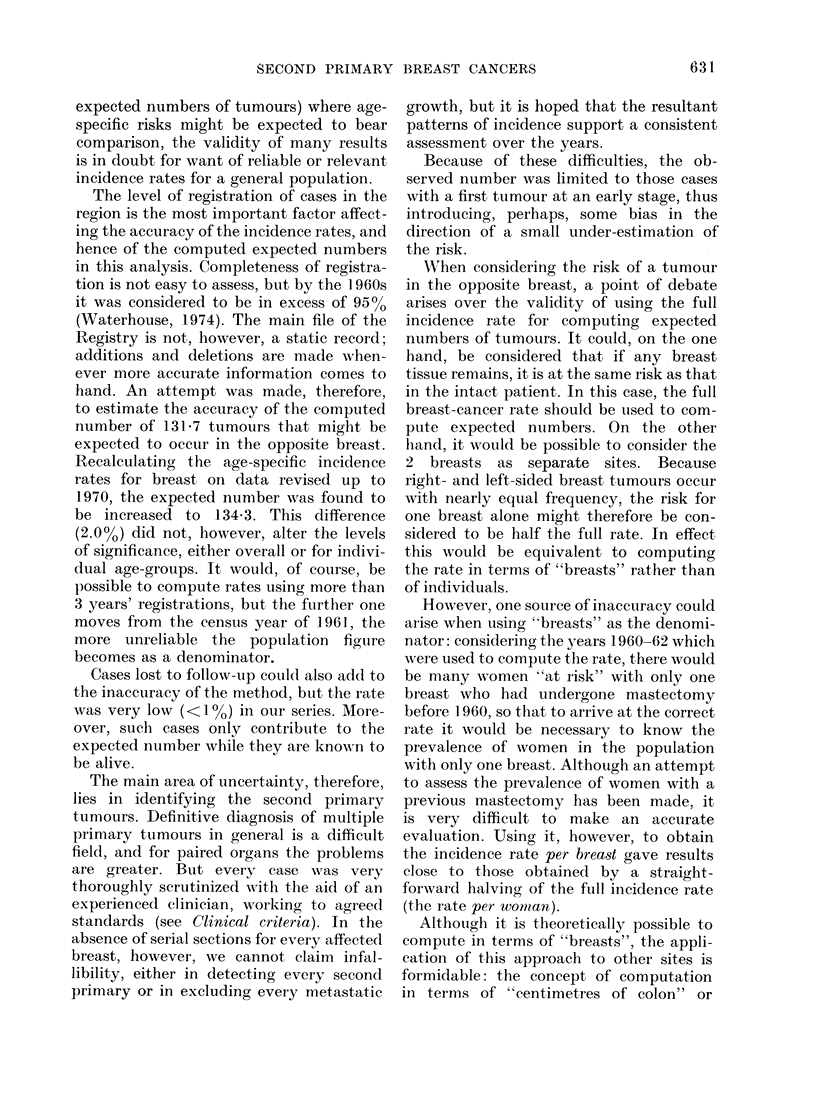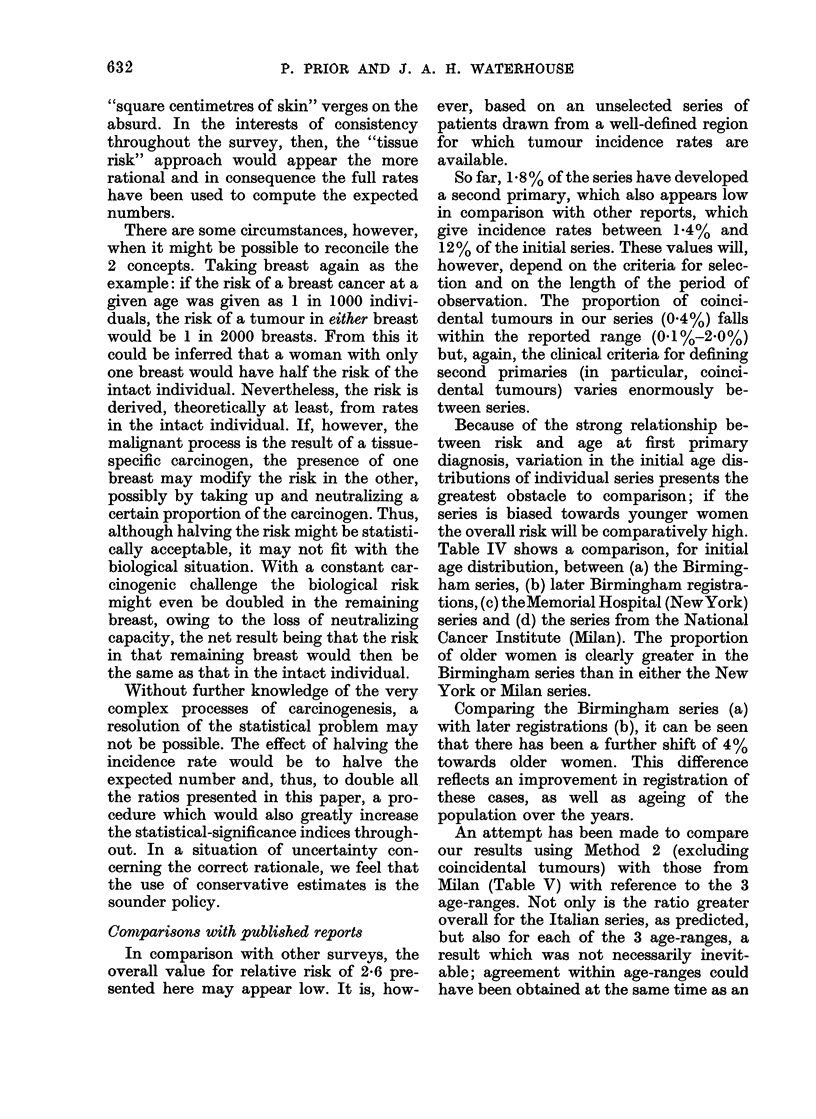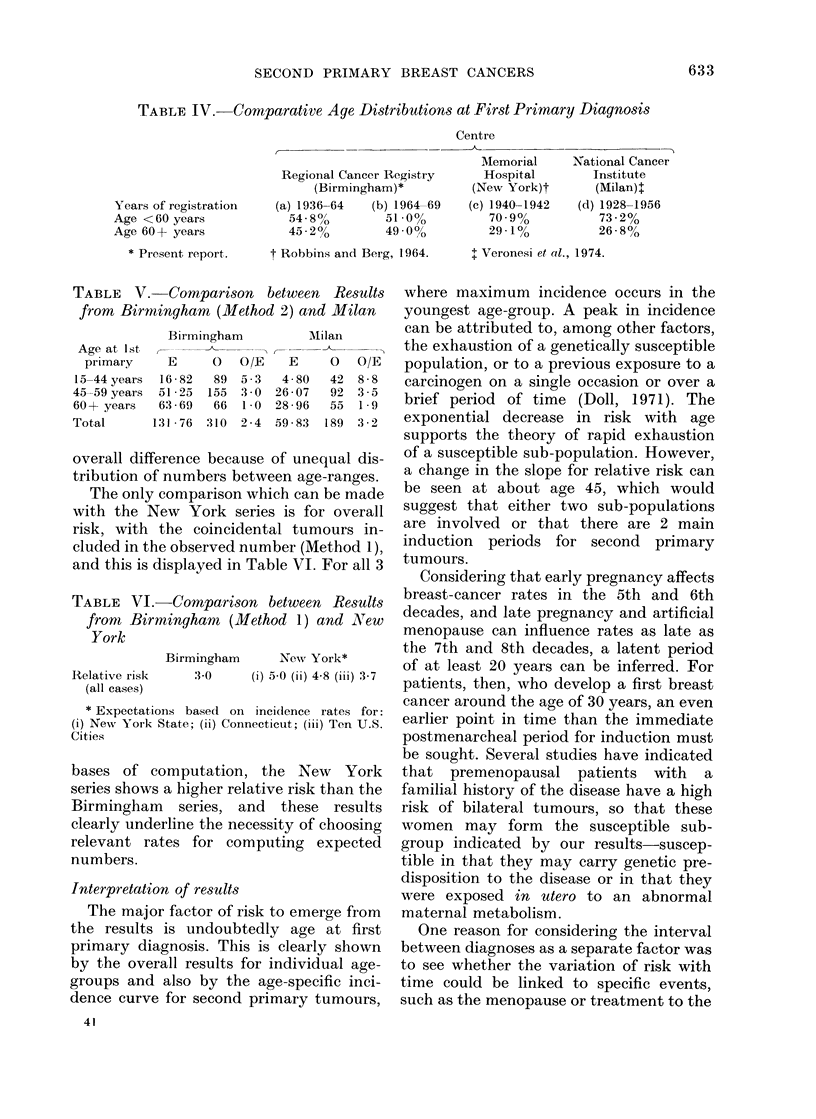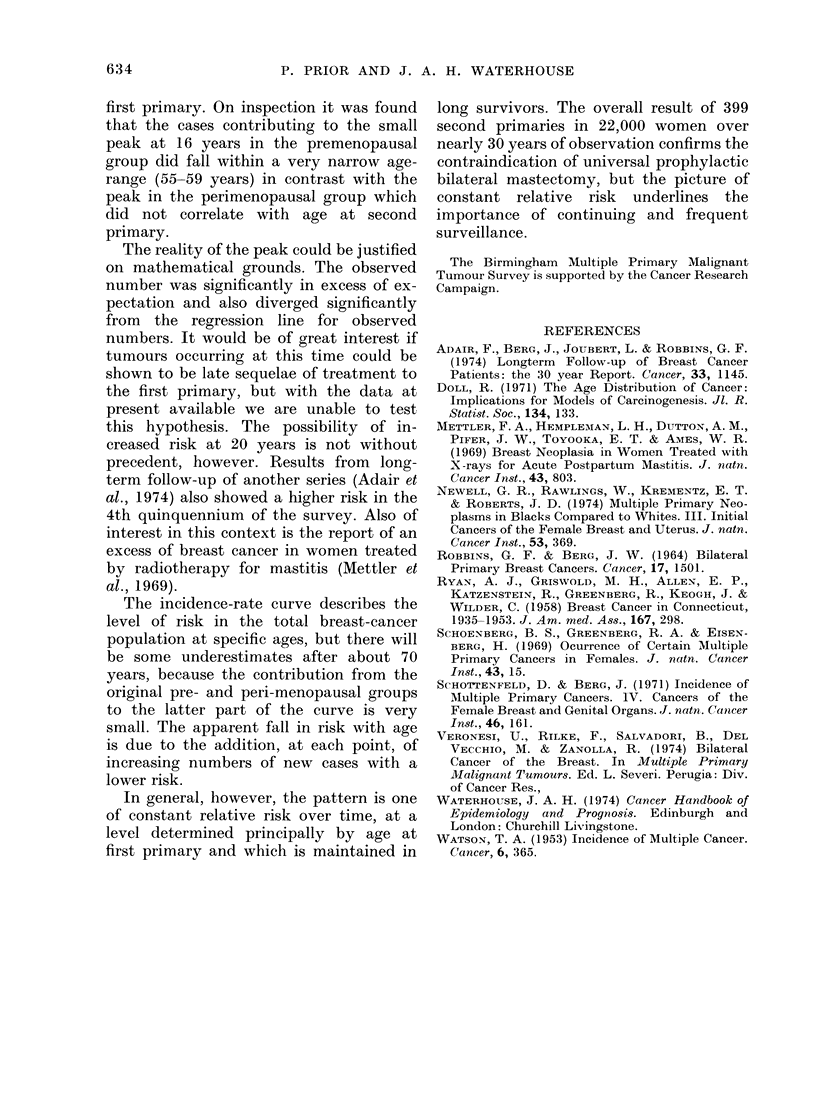# Incidence of bilateral tumours in a population-based series of breast-cancer patients. I. Two approaches to an epidemiological analysis.

**DOI:** 10.1038/bjc.1978.92

**Published:** 1978-04

**Authors:** P. Prior, J. A. Waterhouse

## Abstract

This paper gives the incidence in the Birmingham Regional Cancer Registry (England) of a second primary tumour in the contralateral breast among nearly 22,000 patients registered with a first primary in the breast between the years 1936 and 1964. The results, based on more than 90,000 women-years at risk and 399 second primary tumours, are presented with reference to 2 methods of analysis. In assessing risk, the principal factors investigated were age at first and second primary diagnoses and the interval between diagnoses. The results are discussed in terms of current aetiological hypotheses. On the basis of a method which included coincidental tumours, the overall risk of a tumour in the contraleteral breast was found to be 3.0 times that in the general population of a first primary. The corresponding risks for 3 main age-ranges (at the time of diagnosis of the first primary tumour) were 5.6 (ages 15-44 years), 3.7 (45--59 years) and 1.8 (60+ years). When coincidental tumours were excluded from the analysis, the relative risk was found to be 2.4 overall and 5.3, 3.0 and 1.0 for the 3 age-ranges, respectively. The level of risk was negatively correlated with age at first primary and the relative risk remained substantially constant over time.


*Br. J. Cancer* (1978) **37**, 620

### INCIDENCE OF BILATERAL TUMOURS IN A POPULATION-BASED SERIES OF BREAST-CANCER PATIENTS. I. TWO APPROACHES TO AN EPIDEMIOLOGICAL ANALYSIS

P. PRIOR AND J. A. H. WATERHOUSE

*From the Birmingham Regional Cancer Registry, Queen Elizabeth Hospital, Birmingham, and The Department of Social Medicine, University of Birmingham*

Received 19 August 1977 Accepted 18 January 1978

**Summary.—This paper gives the incidence in the Birmingham Regional Cancer Registry (England) of a second primary tumour in the contralateral breast among nearly 22,000 patients registered with a first primary in the breast between the years 1936 and 1964. The results, based on more than 90,000 woman-years at risk and 399 second primary tumours, are presented with reference to 2 methods of analysis. In assessing risk, the principal factors investigated were age at first and second primary diagnoses and the interval between diagnoses. The results are discussed in terms of current aetiological hypotheses.**

**On the basis of a method which included coincidental tumours, the overall risk of a tumour in the contralateral breast was found to be 3·0 times that in the general population of a first primary. The corresponding risks for 3 main age-ranges (at the time of diagnosis of the first primary tumour) were 5·6 (ages 15–44 years), 3·7 (45–59 years) and 1·8 (60+ years). When coincidental tumours were excluded from the analysis, the relative risk was found to be 2·4 overall and 5·3, 3·0 and 1·0 for the 3 age-ranges, respectively. The level of risk was negatively correlated with age at first primary and the relative risk remained substantially constant over time.**

DESPITE the extensive literature relating to the occurrence of bilateral breast cancer, no firm conclusion about the magnitude of the risk of a new primary tumour in the contralateral breast can yet be drawn because, in the main, reports are based on small selected clinical or necropsy series for which no general comparative figures are available. A knowledge of absolute and differential rates of second primary tumours is not only of great clinical importance for the future management of the breast cancer patient, but might give some clues to the aetiology of the disease itself. This paper is concerned with establishing the incidence of second primary tumours in the contralateral breast in a series of breast-cancer patients drawn from a known general population. The relationship between risk and age is also investigated.

Over the years it has become clear that any reliable estimations must be based on large quantities of accurate data which are at least representative of a known population. The accepted approach to assessment now is to select a large series of breast-cancer patients who have been followed up for many years, to express their survival in terms of patient-years at risk, to compute the number of second primary tumours which might be expected to occur during a pre-set period of time by applying age-specific incidence rates to these years of survival, and to compare the expected number of tumours with that observed, by means of suitable tests of significance.

### *Previous studies*

In the following summary of reports concerning bilateral breast cancer the above method has been used with some modifications and with varying degrees of comparability, but in each case a serious attempt has been made to determine expectations on the basis of incidence rates for a general population; in each case, a large series of patients was available. Even so, the risk of a primary tumour in the opposite breast was found to vary between 1·8 and 5·0 times that of a first primary in the general population.

For a series of over 8000 patients, registered at the Connecticut Cancer Record Registry between 1935 and 1951, an expectation of 51 second primaries was computed, using State incidence rates for the relevant years, in comparison with the observed number of 272. The ratio of *observed* to *expected* was 5·0, but no further details of methodology were reported (Ryan *et al.*, 1958).

At the Memorial Hospital, New York, 1458 women treated by radical mastectomy between the years 1940 and 1943 developed 91 second primaries over a 20-year follow-up. When expectation was based on rates from the Ten Cities Survey, the ratio (*observed: expected*) was found to be 3·7, whereas State incidence rates for Connecticut and New York gave values of 4·8 and 5·0 respectively. These figures clearly underline the importance of selecting an appropriate general population for assessment (Robbins and Berg, 1964).

Data from the hospital-based registry of the Memorial Sloan–Kettering Cancer Center provided a series of 9792 breast-cancer patients found over a period of 13 years. On the basis of New York State incidence rates, 55 second primary breast tumours were expected. Of the 306 cases observed, 58 (19%) were classified as synchronous tumours, and for the remaining 248 the ratio (248/55) was 4·5 (Schottenfeld and Berg, 1971).

An apparent ethnic difference was reported by the Charity Hospital of Louisiana Tumour Registry; the ratio of observed to expected numbers was shown to be 1·8 for white and 3·3 for black women. Here again the expectations were based on the Ten Cities Survey which provided age, site and race-specific rates (Newell *et al.*, 1974).

Among a series of 6986 patients admitted to the National Cancer Institute of Milan, 45 women developed synchronous tumours and 189 consecutive tumours in the opposite breast. Only the latter were compared with the expected number of 59·8, the ratio being 3·15. Expectations were computed from rates supplied by the Cancer Registry of Piemonte and Valle d'Aosta (Veronesi *et al.*, 1974).

*Background to the Birmingham survey*

The investigation presented here forms part of the Multiple Primary Tumour Survey undertaken at the Birmingham Regional Cancer Registry. The Birmingham Region comprises 5 counties with a total population of 5 millions and it is well-defined geographically and administratively. Also, covering both urban and rural areas, it is representative of the whole country.

Registration of cancer cases was started in the Birmingham Region as far back as 1936, and was extended rapidly to cover the whole of the region. By 1961, the level of registration was considered to be high enough (more than 95%) to compute accurate incidence rates for the regional population. The results presented here are, therefore, based on cancer registry data and, as such, may suffer from some of the disadvantages inherent in any large body of information (*e.g.* the multiplicity of standards for diagnosing, treating, classifying and reporting tumours). Also, standards from individual sources change with time in the light of knowledge gained over the years.

Nevertheless, the data available are population-based. By selecting breast as the first-primary site, a large series was obtained, comprising nearly 22,000 patients registered between 1936 and 1964 and followed up regularly thereafter. In contrast with patients with more deep-seated tumours, breast cancer patients experienced a relatively long period of survival (overall some 42·6% of patients survive for 5 years (Waterhouse, 1974)) which allows more chance of observing second primary tumours. Over a maximal period of 29 years the loss to follow-up was less than 1% of all cases. Although for the purposes of this analysis the period of observation was terminated in 1965, it is worth noting that the first four 40-year survivors were recorded during 1976–77.

For reason of large numbers again, breast was chosen in the first instance as the second primary site. In a preliminary survey of all multiple primary tumours known to have occurred before the end of 1959, bilateral breast tumours accounted for 9% of the total, when multiple skin tumours were excluded.

In the absence of serial histological sections for every breast tumour, we have been unable to assess the incidence of multifocal tumours in the same breast. The analysis has therefore been limited to primary tumours in the opposite breast. This approach is in accordance with registration procedure; only very rarely are 2 registrations made for the same breast, when for example a sarcoma coexists with a carcinoma.

Starting therefore with a justifiable degree of confidence in the data, we ventured to hope that the advantages that accrue from information of this quality and quantity would far outweigh the above disadvantages.

### METHOD AND MATERIALS

*Source of data.*—The series comprised 21,967 female breast-cancer patients, registered at the Birmingham Regional Cancer Registry between 1936 and 1964. The only exclusions were registrations for the second primary breast tumour, because these patients could not be considered to be "at-risk" for another breast tumour, and another 14 cases, for whom the age at first diagnosis was unknown, which were excluded because they could not be admitted to the age-specific computations. The overall estimation of risk for a second primary tumour in breast, therefore, refers to a total population of breast-cancer patients and not to a selected sample.

*Data processing.*—Data for all patients were held primarily on punched cards. Taking the 1965 anniversary date of their entry as the closing point to the survey, all cards were updated to this time or to death if this occurred earlier. The records were then transferred to magnetic tape and finally to a computer file store for more rapid access.

*Level of tracing.*—Follow-up information was incomplete for only 165 patients (0·75% or 1 patient per 545 patient-years), half of these being registered during 1936–1950, a period (covering the war years) when tracing was a little more difficult. Nevertheless, some of these cases are even now being recovered through death certificate notifications. For this reason, many of the later registrations with only 1 or 2 years of observation missing are not considered irrevocably lost to the survey.

In the case of a patient not traced to the requisite closing date of the survey, survival was taken up to the last year that she was known to be alive.

*Person-years at risk.*—The survival experienced by the series was expressed as a two-dimensional array of person-years at risk in terms of age-group at, and years of survival from, the time of first primary diagnosis, up to a maximum of 29 years.

*Incidence rate.*—Age-specific incidence rates for breast cancer were computed from the mean number of breast registrations for the Birmingham Region between 1960 and 1962, together with population figures for the Region obtained from the Registrar General's 1961 Population Census.

*Expected number.*—The general approach to the computation of expected numbers was as follows: linear interpolation between the age-specific incidence rates for breast cancer for 5-year age-groups was carried out to give an incidence rate for individual years of age. The rates were then applied to the appropriate yearly elements of the array of person-years at risk, and the expected number of second primary breast tumours to occur during the period of observation, was computed. Modifications to this general approach are described in more detail below.

*Observed number.*—The second primary tumours which developed in the series within the defined period were ascertained by searching the Registry's files meticulously, not only for directly linked records, but also for those cases for whom there was any reference to malignancy in or treatment to the opposite breast. The clinical records for all these patients were then reassessed to confirm their eligibility for inclusion in the survey. Apart from those cases excluded for clinical reasons (see below), more than 100 other cases were not included because either the first primary was not registered in the Birmingham region (and in consequence the patient was not represented in the years at risk) or because the diagnosis of either primary fell outside the time limits of the period of observation. Second primary tumours diagnosed outside the region, but within the prescribed time limits, were included in the analysis, because such patients contributed to the expected numbers of tumours.

*Clinical criteria.*—One disadvantage in selecting paired organs for this type of survey is that difficulty might be experienced in assessing the status of the second growth. The tumours may occur close together in time and, arising in the same type of tissue, they may be of similar histology. For these sites, therefore, the criteria used to assess the primary status of tumours to be included in the survey must be applied rigorously.

In the simplest situation, each breast would show a discrete tumour with or without extension to the ipsilateral axillary nodes. If, however, distant metastases were present either before or at the time of diagnosis of the second growth, the latter was not included as a second primary. Thus, among definite bases for exclusions were: fixed axillary nodes at the time of the first tumour; positive supraclavicular nodes; and skin nodules or distant metastases discovered before or at the same time as the second tumour. Subsequent tumours in the inner quadrant or in the axillary tail were only included with extreme caution. Even so, it may be that some metastatic tumours have been included inadvertently, but they would be offset to some extent by those cases for which the first tumour, or possibly both tumours, were at an advanced stage when the patient was first examined, and were therefore excluded on the basis of the clinical criteria, but which might in fact have been separate primaries.

In addition to the clinical picture, histological reports were available for 93% of first primary tumours and 87% of second tumours. In the early years of the Registry, copies of pathology reports were not always forthcoming, but after 1953 histological slides were reviewed centrally by one pathologist to confirm the diagnosis before registration. When no histological report was available, such as when the only treatment was by radiotherapy or hormones, the case was included if the clinical criteria were satisfied. In addition, the opinion of the reporting clinician and also the general clinical picture was considered (*e.g.*, radical treatment may have been withheld for reasons of age or concurrent disease). If insufficient information was available the case was excluded. All cases were, however, reviewed centrally by one consultant, and the intention throughout was to err in the direction of discretion rather than exaggeration so that the results would represent real effects.

*Statistical significance levels.*—On the basis that second primary tumours are relatively rare events in time, the Poisson distribution has been used to determine the significance of the difference between observed and expected numbers of second primary tumours. Taking the expected number as the mean of the distribution, the probability of the observed number or more occurring by chance was computed. When combining sub-groups, for means greater than 50 the significance was determined from the Normal Deviate, on the assumption that, for means of this order, the Poisson can be sufficiently represented by the Normal distribution with variance equal to the mean. The results are presented in terms of the three conventional levels of significance.

*Coincidental tumours.*—A further problem in methodology was posed by the cluster of coincidental (or synchronous) tumours, which comprised 23% of all the observed cases. In the Birmingham survey, they were defined as those tumours diagnosed at the same time as, or within one month of, the first primary. The question was whether or not they were statistically valid inclusions in the observed number of the analysis. In published surveys, the statistical treatment of these tumours has been varied. For the purposes of comparison, therefore, the results of this survey will be presented here in 2 ways: Method 1 includes all the coincidental tumours in the observed number, leaving expectation, computed in the conventional way, unaltered; Method 2 excludes all coincidental tumours apart from those which might be attributed to the first month of the first year of the survey, again leaving the expected number unaltered.

### RESULTS

### *Analysis by Method* 1

Overall, 399 cases of a second primary tumour in the opposite breast were observed in the series, for an expectation of 132, the risk being, therefore, 3·0 times that of the general population for a first primary at this site. The result was based on 91,233 woman-years at risk (WYR), thus giving an actual incidence of 437/100,000 WYR, in comparison with an expectation of 145/100,000 WYR. Because the period of observation was variable, namely 1 to 29 years according to the year of entry into the series, more tumours will undoubtedly arise in the future, so that the proportion of patients (1·8%) who have already developed a second tumour is a figure of only transient interest whereas, for this particular series, the 93 coincidental tumours (0·42%) which were observed represent a final evaluation.

*Age at first primary diagnosis.*—Summing the results over the whole period of observation, observed and expected numbers for individual 5-year age-groups are displayed in Table I, age at the time of diagnosis of the first primary being the point of reference. It can be seen that the observed tumours are in excess of expectation at a highly significant level ($P < 0{\cdot}001$) for patients 20 to 59 years of age at the time of first primary diagnosis. In patients over 60 years of age, although there is still some excess, the levels of significance are more variable. Fig. 1 shows that relative risk (observed/expected) is negatively associated with age at first primary diagnosis.

*Menopausal status.*—In the absence of complete data for date of menopause, the age groups were combined into 3 main age-ranges, which were selected to cover, broadly speaking, the phases of menopausal status (*viz.* premenopausal (15–44 years), perimenopausal (45–59 years) and postmenopausal (60+ years)). Second primary tumours were significantly in excess in each age range, but again, the ratio of observed to expected number decreased with age (Table I), the relative risk in the postmenopausal group being only one-third that of the premenopausal patients.

TABLE I.—*Relative Risk of a Second Primary Breast Tumour in Relation to Age at First Primary Diagnosis* (*Method* 1)

| Age group | E | O | O/E | P | Age range | E | O | O/E | P |
|---|---|---|---|---|---|---|---|---|---|
| 15–19† | 0·00 | 0 | — | — | | | | | |
| 20–24 | 0·01 | 3 | 300·0 | *** | | | | | |
| 25–29 | 0·15 | 6 | 40·0 | *** | | | | | |
| 30–34 | 0·88 | 9 | 10·2 | *** | | | | | |
| 35–39 | 4·09 | 28 | 6·9 | *** | | | | | |
| 40–44 | 11·70 | 49 | 4·2 | *** | Premenopausal 15–44 | 16·82 | 95 | 5·6 | *** |
| 45–49 | 18·36 | 79 | 4·3 | *** | | | | | |
| 50–54 | 16·37 | 59 | 3·6 | *** | | | | | |
| 55–59 | 16·51 | 52 | 3·1 | *** | Perimenopausal 45–59 | 51·25 | 190 | 3·7 | *** |
| 60–64 | 17·83 | 33 | 1·9 | ** | | | | | |
| 65–69 | 16·90 | 35 | 2·1 | *** | | | | | |
| 70–74 | 13·09 | 24 | 1·8 | * | | | | | |
| 75–79 | 9·13 | 10 | 1·1 | — | | | | | |
| 80–84 | 4·62 | 10 | 2·2 | * | | | | | |
| 85–89 | 1·61 | 2 | 1·2 | — | | | | | |
| 90–94 | 0·37 | 0 | — | — | | | | | |
| 95–99+ | 0·14 | 0 | — | — | Postmenopausal 60+ | 63·69 | 114 | 1·8 | *** |
| Total | | | | | | 131·76 | 399 | 3·0 | *** |

† Only 1 patient at risk

E: Expected, O: Observed — number of second primary tumours.

FIG. 1.—Relative risk of 2nd primary breast tumours in relation to age at diagnosis of the 1st primary tumour in breast (Method 1).

*Interval between diagnoses.*—Summing over age groups, the results have also been analysed in relation to the interval of time between the diagnoses of first and second primary tumours. They are tabulated by individual years and also by quinquennia (Table II). The difference between the observed number and expectation was highly significant ($P < 0{\cdot}001$) up to Year 4; thereafter, individual values were significantly different from expectation but at varying levels.

In terms of quinquennia, there was a highly significant ($P < 0{\cdot}001$) excess of tumours in Periods 1, 2 and 3. For Period 4, although the observed number was nearly double the expectation, the difference did not reach the 5% level of significance. A significant ($P < 0{\cdot}05$) excess was again found in Period 5; no tumours were observed in the final 4 years.

TABLE II.—*Relative Risk of a Second Primary Breast Tumour in Relation to Interval Between First and Second Primary Diagnoses (Method 1)*

| Interval (years from 1st primary) | Annual | | | | Quinquennial | | | |
|---|---|---|---|---|---|---|---|---|
| | E | O | O/E | P | E | O | O/E | P |
| 1 | 27·16 | 146 | 5·4 | *** | | | | |
| 2 | 20·24 | 54 | 2·7 | *** | | | | |
| 3 | 15·59 | 56 | 3·6 | *** | 85·26 | 301 | 3·5 | *** |
| 4 | 12·33 | 27 | 2·2 | *** | | | | |
| 5 | 9·94 | 18 | 1·8 | * | | | | |
| 6 | 8·07 | 21 | 2·6 | *** | | | | |
| 7 | 6·59 | 11 | 1·7 | — | | | | |
| 8 | 5·32 | 11 | 2·1 | * | 28·08 | 59 | 2·1 | *** |
| 9 | 4·37 | 12 | 2·7 | ** | | | | |
| 10 | 3·73 | 4 | 1·1 | — | | | | |
| 11 | 3·18 | 9 | 2·8 | * | | | | |
| 12 | 2·71 | 6 | 2·2 | — | | | | |
| 13 | 2·32 | 3 | 1·3 | — | 11·76 | 26 | 2·2 | *** |
| 14 | 1·94 | 2 | 1·0 | — | | | | |
| 15 | 1·61 | 6 | 3·7 | ** | | | | |
| 16 | 1·32 | 1 | 0·8 | — | | | | |
| 17 | 1·11 | 2 | 1·8 | — | | | | |
| 18 | 0·93 | 1 | 1·1 | — | 4·73 | 8 | 1·7 | — |
| 19 | 0·77 | 1 | 1·3 | — | | | | |
| 20 | 0·60 | 3 | 5·0 | * | | | | |
| 21 | 0·48 | 4 | 8·3 | *** | | | | |
| 22 | 0·39 | 1 | 2·6 | — | | | | |
| 23 | 0·31 | 0 | — | — | 1·60 | 5 | 3·1 | * |
| 24 | 0·23 | 0 | — | — | | | | |
| 25 | 0·19 | 0 | — | — | | | | |
| 26 | 0·14 | 0 | — | — | | | | |
| 27 | 0·10 | 0 | — | — | 0·33 | 0 | — | — |
| 28 | 0·06 | 0 | — | — | | | | |
| 29 | 0·03 | 0 | — | — | | | | |
| Total | | | | | 131·76 | 399 | 3·0 | *** |

FIG. 2.—Observed (——, 3-point moving average) and expected (– – –) numbers of 2nd primary breast tumours by year from 1st primary diagnosis. (Method 1).

Fig. 2 displays these results in the form of a graph. The observed numbers are drawn as a 3-point moving average to allow for the meaningful comparison between integer (observed number) and real (expected number) values. In spite of the apparent peak in observed risk at about 20 years, the line for observed numbers shows essentially the same rate of fall as that for expected numbers ($b_O = -0{\cdot}084$, $b_E = -0{\cdot}083$, s.e. (Diff.) $= 0{\cdot}0063$, $P \gg 0{\cdot}05$). When the breast-cancer population is viewed as a whole, the relative risk appears constant over time and is 2·4 times that for the general population.

Although the 3-point moving average smoothed many of the irregularities, the peak at 20 years was not eliminated. Although the point lies outside the 95% confidence limits of the observed regression line and is significantly in excess of expectation, more data for this period will be required to ensure that the peak is not an artefact of small numbers or of follow-up procedures which highlight periods of 5, 10, 15 and 20 years.

*Interval between diagnosis in relation to age at first primary.*—The overall result presented above does, however, represent an aggregation of effects for all age-groups and, when observed and expected numbers are broken down to the 3 age-ranges, differences in the pattern of diagnostic interval become apparent. Figs. 3(a), (b)

Fig. 3(a)

Fig. 3(b)

Fig. 3(c)

FIG. 3.—Observed (——, 3-point moving average) and expected (– – –) number of 2nd primary breast tumours by year from 1st primary diagnosis for the 3 age-ranges: (a). 15–44 years, (b). 45–59 years, (c). 60+ years. (Method 1).

and (c) present the results for these groups, with the observed number again shown as a 3-point moving average, to demonstrate more clearly the trend in the development of second primary tumours.

*Premenopausal group.*—At the beginning of the period, it can be seen (Fig. 3(a)) that the observed number is 10 times expectation, but the excess decreases until, at about Year 12, it is close to expectation. There is some evidence of a later peak at Year 16, but the numbers involved by this time are very small. Regression analysis suggests that the risk is falling over time for this group ($b_O = 0{\cdot}098$, $b_E = 0{\cdot}054$, s.e. (Diff.) $= 0{\cdot}0077$, $P < 0{\cdot}001$).

*Perimenopausal group.*—Although the general pattern for this group (Fig. 3(b)) is similar to that of the younger patients, the observed risk at the start of the survey is only 4·5 times expectation and falls more slowly, reaching expectation at Year 17. For this group, the peak at about 20 years is more pronounced, although again the numbers involved are small and regression analysis indicates a constant relative risk ($b_O = 0{\cdot}072$, $b_E = 0{\cdot}075$, s.e. (Diff.) $= 0{\cdot}0084$, $P$ not significant).

*Postmenopausal group.*—Initially, the observed number for this group is twice expectation (Fig. 3C). In subsequent years it follows the expected value more closely, although always lying above it, giving an overall relative risk of 1·8. For this group a small divergence of the regression lines over time was observed ($b_O = 0{\cdot}094$, $b_E = 0{\cdot}105$, s.e. (Diff.) $= 0{\cdot}005$, $P < 0{\cdot}05$).

Although the picture of constant relative risk for the whole series is an acceptable mathematical result of the parallelism shown in Fig. 2, it is compounded from differing patterns for the three age-ranges.

*Age at second primary diagnosis.*—Previous reports (Watson, 1953; Schoenberg, Greenberg and Eisenberg, 1969) grouped the woman-years at risk in terms of age-specific experience (forfeiting in the process the factor of "age at first primary"). When the data for the Birmingham survey were re-grouped in a similar way, it was shown (Fig. 4) that the second primary occurred at a relatively early age (median age 59 years) compared with expectation (median age 67·5 years).

Using the same technique it was possible to construct an age-specific incidence rate of secondary primary tumours in the cancer population, which has been compared with the rate of first primary breast cancer in the general population in Fig. 5. There would appear to be a change in slope around the age of 45 years, similar but in the opposite direction to that of first primaries.

### *Analysis by Method 2*

Method 2 was adopted mainly to allow for comparison with other published series which excluded coincidental tumours from their analyses. Basically, the approach was to repeat all the computations described under Method 1, but with the coincidental tumours removed from the original observed number. It would follow, from our definition of coincidental tumours, that some tumours which could be

FIG. 4.—Observed and expected numbers and relative risk of 2nd primary breast tumours in relation to age at 2nd primary diagnosis (Method 1).

ascribed to the first month of the survey would have been eliminated with those diagnosed at the same time as the first primary. When cumulative numbers of observed tumours were plotted by month for the first year, it was found that a linear graph was obtained (Fig. 6) so by taking the mean number per month during the interval 0 years 1 month and 0 years 11 months, a value for the first month (0 years 0 months) was estimated. The 4 tumours which were included for the first month were allocated to age-groups 40–44, 45–49, 50–54, 55–59, on the basis of nearest integer.

*Age at first primary diagnosis.*—Table III displays the results given by this method for 5-year age-groups, and shows that below the age of 60 years the observed number of tumours is still in excess of expectation at a highly significant level ($P < 0{\cdot}001$). In contrast to Method 1, for patients aged 60 years or more, only one group (65–69 years) shows a significant result ($P < 0{\cdot}05$). For the pre- and peri-menopausal age-ranges the order of significance ($P < 0{\cdot}001$) is unchanged, although the relative risk is reduced. But for the postmenopausal patients, the overall relative risk is now 1·0 (*i.e.* the observed number is close to expectation).

A comparison between Methods 1 and 2 for relative risk, by age at first primary diagnosis (Fig. 7), shows again that the main differences lie in the results for the postmenopausal patients.

*Interval between diagnoses.*—Adjustment of the observed number by Method 2 changes the result of the analysis in terms

FIG. 5.—Age-specific incidence rates (N/$10^5$ WYR) of 2nd primary tumours (——) in comparison with those for 1st primary breast tumours (– – –) (Method 1).

FIG. 6.—Cumulative observed number of 2nd primary breast tumours diagnosed during the 1st year of observation.

TABLE III.—*Relative Risk of a Second Primary Tumour in Breast in Relation to Age at First Primary* (*Method* 2)

| Age group | E | O | O/E | P | E | O | O/E | P |
|---|---|---|---|---|---|---|---|---|
| 15–19† | 0·00 | 0 | — | — | | | | |
| 20–24 | 0·01 | 1 | 100·0 | * | | | | |
| 25–29 | 0·15 | 5 | 33·3 | *** | | | | |
| 30–34 | 0·88 | 9 | 10·2 | *** | | | | |
| 35–39 | 4·09 | 27 | 6·6 | *** | Premenopausal | | | |
| 40–44 | 11·70 | 47 | 4·0 | *** | 16·82 | 89 | 5·3 | *** |
| 45–49 | 18·36 | 66 | 3·6 | *** | | | | |
| 50–54 | 16·37 | 52 | 3·2 | *** | Perimenopausal | | | |
| 55–59 | 16·51 | 37 | 2·2 | *** | 51·25 | 155 | 3·0 | *** |
| 60–64 | 17·83 | 18 | 1·1 | — | | | | |
| 65–69 | 16·90 | 25 | 1·5 | * | | | | |
| 70–74 | 13·09 | 14 | 1·1 | — | | | | |
| 75–79 | 9·13 | 2 | 0·2 | — | | | | |
| 80–84 | 4·62 | 7 | 1·5 | — | | | | |
| 85–89 | 1·61 | 0 | — | — | | | | |
| 90–94 | 0·37 | 0 | — | — | Postmenopausal | | | |
| 95+ | 0·14 | 0 | — | — | 63·69 | 66 | 1·0 | — |
| Total | | | | | 131·76 | 310 | 2·4 | *** |

Fig. 7.—Comparison between Method 1 and Method 2 for relative risk in terms of age at 1st primary.

Fig. 8.—Comparison between Method 1 and 2 for relative risk in terms of age at 2nd primary.

of interval between diagnoses in 3 details: the relative risks for

(i) the first year = 57/27·16 = 2·1
(ii) the first quinquennium = 212/85·26 = 2·5
(iii) overall = 310/131·76 = 2·4.

In all other respects the results given in Table II remain the same for each method of analysis.

*Age at second primary diagnosis.*—Exclusion of the coincidental tumours from this analysis reduced the risk at each age, but before the age of 60 years the risk remained significantly high. After age 60, the observed number approached expectation more closely. The analyses for Method 1 and 2 are compared in Fig. 8.

### DISCUSSION

### *Methodology*

A substantial series of patients, followed for many years, is required to establish the absolute risk of second primary tumours. Even so, a large quantity of data does not necessarily ensure freedom from bias, for example patients drawn from one locality may be markedly different in many respects from the totality of cancer patients in contiguous areas.

Valid comparisons between individual series are also difficult to make because, apart from hospital selection which could introduce bias due to socio-economic status, ethnic origin, occupation or even the type of treatment required, each series will start with differing age distributions and will experience varying periods of observation and patterns of mortality. Final results will reflect such variation, whether they are presented in terms of percentage or as numbers of tumours per $10^5$ person years at risk. For example, a large series followed for only 5 years could show a different level of risk from a smaller series followed for 20 years, although the number of WYR might be the same in each instance.

Even when the more sophisticated approach to analysis is used (computing expected numbers of tumours) where age-specific risks might be expected to bear comparison, the validity of many results is in doubt for want of reliable or relevant incidence rates for a general population.

The level of registration of cases in the region is the most important factor affecting the accuracy of the incidence rates, and hence of the computed expected numbers in this analysis. Completeness of registration is not easy to assess, but by the 1960s it was considered to be in excess of 95% (Waterhouse, 1974). The main file of the Registry is not, however, a static record; additions and deletions are made whenever more accurate information comes to hand. An attempt was made, therefore, to estimate the accuracy of the computed number of 131·7 tumours that might be expected to occur in the opposite breast. Recalculating the age-specific incidence rates for breast on data revised up to 1970, the expected number was found to be increased to 134·3. This difference (2.0%) did not, however, alter the levels of significance, either overall or for individual age-groups. It would, of course, be possible to compute rates using more than 3 years' registrations, but the further one moves from the census year of 1961, the more unreliable the population figure becomes as a denominator.

Cases lost to follow-up could also add to the inaccuracy of the method, but the rate was very low ($<1\%$) in our series. Moreover, such cases only contribute to the expected number while they are known to be alive.

The main area of uncertainty, therefore, lies in identifying the second primary tumours. Definitive diagnosis of multiple primary tumours in general is a difficult field, and for paired organs the problems are greater. But every case was very thoroughly scrutinized with the aid of an experienced clinician, working to agreed standards (see *Clinical criteria*). In the absence of serial sections for every affected breast, however, we cannot claim infallibility, either in detecting every second primary or in excluding every metastatic growth, but it is hoped that the resultant patterns of incidence support a consistent assessment over the years.

Because of these difficulties, the observed number was limited to those cases with a first tumour at an early stage, thus introducing, perhaps, some bias in the direction of a small under-estimation of the risk.

When considering the risk of a tumour in the opposite breast, a point of debate arises over the validity of using the full incidence rate for computing expected numbers of tumours. It could, on the one hand, be considered that if any breast tissue remains, it is at the same risk as that in the intact patient. In this case, the full breast-cancer rate should be used to compute expected numbers. On the other hand, it would be possible to consider the 2 breasts as separate sites. Because right- and left-sided breast tumours occur with nearly equal frequency, the risk for one breast alone might therefore be considered to be half the full rate. In effect this would be equivalent to computing the rate in terms of "breasts" rather than of individuals.

However, one source of inaccuracy could arise when using "breasts" as the denominator: considering the years 1960–62 which were used to compute the rate, there would be many women "at risk" with only one breast who had undergone mastectomy before 1960, so that to arrive at the correct rate it would be necessary to know the prevalence of women in the population with only one breast. Although an attempt to assess the prevalence of women with a previous mastectomy has been made, it is very difficult to make an accurate evaluation. Using it, however, to obtain the incidence rate *per breast* gave results close to those obtained by a straightforward halving of the full incidence rate (the rate *per woman*).

Although it is theoretically possible to compute in terms of "breasts", the application of this approach to other sites is formidable: the concept of computation in terms of "centimetres of colon" or

"square centimetres of skin" verges on the absurd. In the interests of consistency throughout the survey, then, the "tissue risk" approach would appear the more rational and in consequence the full rates have been used to compute the expected numbers.

There are some circumstances, however, when it might be possible to reconcile the 2 concepts. Taking breast again as the example: if the risk of a breast cancer at a given age was given as 1 in 1000 individuals, the risk of a tumour in *either* breast would be 1 in 2000 breasts. From this it could be inferred that a woman with only one breast would have half the risk of the intact individual. Nevertheless, the risk is derived, theoretically at least, from rates in the intact individual. If, however, the malignant process is the result of a tissue-specific carcinogen, the presence of one breast may modify the risk in the other, possibly by taking up and neutralizing a certain proportion of the carcinogen. Thus, although halving the risk might be statistically acceptable, it may not fit with the biological situation. With a constant carcinogenic challenge the biological risk might even be doubled in the remaining breast, owing to the loss of neutralizing capacity, the net result being that the risk in that remaining breast would then be the same as that in the intact individual.

Without further knowledge of the very complex processes of carcinogenesis, a resolution of the statistical problem may not be possible. The effect of halving the incidence rate would be to halve the expected number and, thus, to double all the ratios presented in this paper, a procedure which would also greatly increase the statistical-significance indices throughout. In a situation of uncertainty concerning the correct rationale, we feel that the use of conservative estimates is the sounder policy.

*Comparisons with published reports*

In comparison with other surveys, the overall value for relative risk of 2·6 presented here may appear low. It is, however, based on an unselected series of patients drawn from a well-defined region for which tumour incidence rates are available.

So far, 1·8% of the series have developed a second primary, which also appears low in comparison with other reports, which give incidence rates between 1·4% and 12% of the initial series. These values will, however, depend on the criteria for selection and on the length of the period of observation. The proportion of coincidental tumours in our series (0·4%) falls within the reported range (0·1%–2·0%) but, again, the clinical criteria for defining second primaries (in particular, coincidental tumours) varies enormously between series.

Because of the strong relationship between risk and age at first primary diagnosis, variation in the initial age distributions of individual series presents the greatest obstacle to comparison; if the series is biased towards younger women the overall risk will be comparatively high. Table IV shows a comparison, for initial age distribution, between (a) the Birmingham series, (b) later Birmingham registrations, (c) the Memorial Hospital (New York) series and (d) the series from the National Cancer Institute (Milan). The proportion of older women is clearly greater in the Birmingham series than in either the New York or Milan series.

Comparing the Birmingham series (a) with later registrations (b), it can be seen that there has been a further shift of 4% towards older women. This difference reflects an improvement in registration of these cases, as well as ageing of the population over the years.

An attempt has been made to compare our results using Method 2 (excluding coincidental tumours) with those from Milan (Table V) with reference to the 3 age-ranges. Not only is the ratio greater overall for the Italian series, as predicted, but also for each of the 3 age-ranges, a result which was not necessarily inevitable; agreement within age-ranges could have been obtained at the same time as an

TABLE IV.—*Comparative Age Distributions at First Primary Diagnosis*

| | Centre | | | |
|---|---|---|---|---|
| | Regional Cancer Registry (Birmingham)* | | Memorial Hospital (New York)† | National Cancer Institute (Milan)‡ |
| Years of registration | (a) 1936–64 | (b) 1964–69 | (c) 1940–1942 | (d) 1928–1956 |
| Age <60 years | 54·8% | 51·0% | 70·9% | 73·2% |
| Age 60+ years | 45·2% | 49·0% | 29·1% | 26·8% |

* Present report. † Robbins and Berg, 1964. ‡ Veronesi *et al.*, 1974.

TABLE V.—*Comparison between Results from Birmingham (Method 2) and Milan*

| Age at 1st primary | Birmingham E | O | O/E | Milan E | O | O/E |
|---|---|---|---|---|---|---|
| 15–44 years | 16·82 | 89 | 5·3 | 4·80 | 42 | 8·8 |
| 45–59 years | 51·25 | 155 | 3·0 | 26·07 | 92 | 3·5 |
| 60+ years | 63·69 | 66 | 1·0 | 28·96 | 55 | 1·9 |
| Total | 131·76 | 310 | 2·4 | 59·83 | 189 | 3·2 |

overall difference because of unequal distribution of numbers between age-ranges.

The only comparison which can be made with the New York series is for overall risk, with the coincidental tumours included in the observed number (Method 1), and this is displayed in Table VI. For all 3 bases of computation, the New York series shows a higher relative risk than the Birmingham series, and these results clearly underline the necessity of choosing relevant rates for computing expected numbers.

TABLE VI.—*Comparison between Results from Birmingham (Method 1) and New York*

| | Birmingham | New York* |
|---|---|---|
| Relative risk (all cases) | 3·0 | (i) 5·0 (ii) 4·8 (iii) 3·7 |

* Expectations based on incidence rates for: (i) New York State; (ii) Connecticut; (iii) Ten U.S. Cities

*Interpretation of results*

The major factor of risk to emerge from the results is undoubtedly age at first primary diagnosis. This is clearly shown by the overall results for individual age-groups and also by the age-specific incidence curve for second primary tumours, where maximum incidence occurs in the youngest age-group. A peak in incidence can be attributed to, among other factors, the exhaustion of a genetically susceptible population, or to a previous exposure to a carcinogen on a single occasion or over a brief period of time (Doll, 1971). The exponential decrease in risk with age supports the theory of rapid exhaustion of a susceptible sub-population. However, a change in the slope for relative risk can be seen at about age 45, which would suggest that either two sub-populations are involved or that there are 2 main induction periods for second primary tumours.

Considering that early pregnancy affects breast-cancer rates in the 5th and 6th decades, and late pregnancy and artificial menopause can influence rates as late as the 7th and 8th decades, a latent period of at least 20 years can be inferred. For patients, then, who develop a first breast cancer around the age of 30 years, an even earlier point in time than the immediate postmenarcheal period for induction must be sought. Several studies have indicated that premenopausal patients with a familial history of the disease have a high risk of bilateral tumours, so that these women may form the susceptible sub-group indicated by our results—susceptible in that they may carry genetic predisposition to the disease or in that they were exposed *in utero* to an abnormal maternal metabolism.

One reason for considering the interval between diagnoses as a separate factor was to see whether the variation of risk with time could be linked to specific events, such as the menopause or treatment to the first primary. On inspection it was found that the cases contributing to the small peak at 16 years in the premenopausal group did fall within a very narrow age-range (55–59 years) in contrast with the peak in the perimenopausal group which did not correlate with age at second primary.

The reality of the peak could be justified on mathematical grounds. The observed number was significantly in excess of expectation and also diverged significantly from the regression line for observed numbers. It would be of great interest if tumours occurring at this time could be shown to be late sequelae of treatment to the first primary, but with the data at present available we are unable to test this hypothesis. The possibility of increased risk at 20 years is not without precedent, however. Results from long-term follow-up of another series (Adair *et al.*, 1974) also showed a higher risk in the 4th quinquennium of the survey. Also of interest in this context is the report of an excess of breast cancer in women treated by radiotherapy for mastitis (Mettler *et al.*, 1969).

The incidence-rate curve describes the level of risk in the total breast-cancer population at specific ages, but there will be some underestimates after about 70 years, because the contribution from the original pre- and peri-menopausal groups to the latter part of the curve is very small. The apparent fall in risk with age is due to the addition, at each point, of increasing numbers of new cases with a lower risk.

In general, however, the pattern is one of constant relative risk over time, at a level determined principally by age at first primary and which is maintained in long survivors. The overall result of 399 second primaries in 22,000 women over nearly 30 years of observation confirms the contraindication of universal prophylactic bilateral mastectomy, but the picture of constant relative risk underlines the importance of continuing and frequent surveillance.

The Birmingham Multiple Primary Malignant Tumour Survey is supported by the Cancer Research Campaign.

### REFERENCES

ADAIR, F., BERG, J., JOUBERT, L. & ROBBINS, G. F. (1974) Longterm Follow-up of Breast Cancer Patients: the 30 year Report. *Cancer*, **33**, 1145.

DOLL, R. (1971) The Age Distribution of Cancer: Implications for Models of Carcinogenesis. *Jl. R. Statist. Soc.*, **134**, 133.

METTLER, F. A., HEMPLEMAN, L. H., DUTTON, A. M., PIFER, J. W., TOYOOKA, E. T. & AMES, W. R. (1969) Breast Neoplasia in Women Treated with X-rays for Acute Postpartum Mastitis. *J. natn. Cancer Inst.*, **43**, 803.

NEWELL, G. R., RAWLINGS, W., KREMENTZ, E. T. & ROBERTS, J. D. (1974) Multiple Primary Neoplasms in Blacks Compared to Whites. III. Initial Cancers of the Female Breast and Uterus. *J. natn. Cancer Inst.*, **53**, 369.

ROBBINS, G. F. & BERG, J. W. (1964) Bilateral Primary Breast Cancers. *Cancer*, **17**, 1501.

RYAN, A. J., GRISWOLD, M. H., ALLEN, E. P., KATZENSTEIN, R., GREENBERG, R., KEOGH, J. & WILDER, C. (1958) Breast Cancer in Connecticut, 1935–1953. *J. Am. med. Ass.*, **167**, 298.

SCHOENBERG, B. S., GREENBERG, R. A. & EISENBERG, H. (1969) Ocurrence of Certain Multiple Primary Cancers in Females. *J. natn. Cancer Inst.*, **43**, 15.

SCHOTTENFELD, D. & BERG, J. (1971) Incidence of Multiple Primary Cancers. IV. Cancers of the Female Breast and Genital Organs. *J. natn. Cancer Inst.*, **46**, 161.

VERONESI, U., RILKE, F., SALVADORI, B., DEL VECCHIO, M. & ZANOLLA, R. (1974) Bilateral Cancer of the Breast. In *Multiple Primary Malignant Tumours.* Ed. L. Severi. Perugia: Div. of Cancer Res.,

WATERHOUSE, J. A. H. (1974) *Cancer Handbook of Epidemiology and Prognosis.* Edinburgh and London: Churchill Livingstone.

WATSON, T. A. (1953) Incidence of Multiple Cancer. *Cancer*, **6**, 365.